# Mid-range theory of the nursing diagnosis Overweight

**DOI:** 10.1590/0034-7167-2023-0372

**Published:** 2024-06-14

**Authors:** Ana Carolina Costa Carino, Renata Marinho Fernandes, Anna Thays Dias Almeida, Cecília Maria Farias de Queiroz Frazão, Caroline Evelin Nascimento Kluczynik, Ana Luisa Brandão de Carvalho Lira

**Affiliations:** I Universidade Federal do Rio Grande do Norte. Natal, Rio Grande do Norte, Brazil; II Universidade Federal de Pernambuco. Recife, Pernambuco, Brazil; III Universidade Federal do Ceará. Fortaleza, Ceará, Brazil

**Keywords:** Nursing Theory, Nursing Diagnosis, Overweight, Adolescent, Young Adult, Teoria de Enfermagem, Diagnósticos de Enfermagem, Sobrepeso, Adolescente, Adulto Jovem, Teoría de Enfermería, Diagnóstico de Enfermería, Sobrepeso, Adolescente, Adulto Joven

## Abstract

**Objective:**

To build a mid-range theory for the nursing diagnosis Overweight in adolescents and young adults.

**Methods:**

A methodological study in the light of the theoretical frameworks of Roy and of Lopes, Silva and Herdman. A total of 3,925 articles were retrieved and assessed using the State of the Art Through Systematic Review software. The final sample consisted of 28 articles.

**Results:**

The findings converged to 3 essential attributes, 13 antecedents, and 7 consequences. A mid-range theory was built consisting of an illustrated diagram, 11 propositions, and 12 causal relationships.

**Final considerations:**

From the creation of the theory, it was possible to better understand the nursing diagnosis Overweight within the context of adolescents and young adults. Understanding nursing phenomena contributes to nursing science’s advancement and strengthening.

## RESUMO

**Objetivo:**

Construir uma teoria de médio alcance para o diagnóstico de enfermagem Sobrepeso em adolescentes e adultos jovens.

**Método:**

Estudo metodológico à luz dos referenciais teóricos de Roy e Lopes, Silva e Herdman. Um total de 3.925 artigos foram identificados e avaliados usando o programa *State of the Art Through Systematic Review*. A amostra final foi composta por 28 artigos.

**Resultados:**

Os achados convergiram para 3 atributos essenciais, 13 antecedentes e 7 consequentes. Foi construída uma teoria de médio alcance composta por um diagrama ilustrado, 11 proposições e 12 relações causais.

**Considerações finais:**

A partir da elaboração da teoria, foi possível compreender melhor o diagnóstico de enfermagem Sobrepeso no contexto de adolescentes e adultos jovens. A compreensão dos fenômenos da enfermagem contribui para o avanço e fortalecimento da ciência da enfermagem.

## RESUMEN

**Objetivo:**

Construir una teoría de rango medio para el diagnóstico de enfermería Sobrepeso en adolescentes y adultos jóvenes.

**Métodos:**

Estudio metodológico a la luz de los marcos teóricos de Roy y de Lopes, Silva y Herdman. Un total de 3.925 artículos fueron recuperados y evaluados utilizando el software *State of the Art Through Systematic Review*. La muestra final fue de 28 artículos.

**Resultados:**

Los resultados convergieron en 3 atributos esenciales, 13 antecedentes y 7 consecuencias. Se construyó una teoría de rango medio compuesta por un diagrama ilustrado, 11 proposiciones y 12 relaciones causales.

**Consideraciones finales:**

A partir de la creación de la teoría, fue posible comprender mejor el diagnóstico de enfermería Sobrepeso en el contexto de adolescentes y adultos jóvenes. La comprensión de los fenómenos de enfermería contribuye para el avance y fortalecimiento de la ciencia de enfermería.

## INTRODUCTION

Mid-range theories (MRT) in nursing are an effective strategy to reduce the distance between clinical practice and teaching. Developing key concepts and measurable variables helps nurses in individualized critical and clinical reasoning, promoting the accuracy of nursing diagnoses^(1, 2)^. However, despite representing the knowledge of the discipline and guiding practice, the development of MRTs worldwide and nationally is still limited. Few studies have addressed mid-range theories in Brazil, causing a theoretical assistance gap in various settings^(3, 4)^.

Given the above, healthcare for people with overweight stands out due to the wide variety of generic meanings of clinical indicators and factors related to this nursing diagnosis (ND), which makes it difficult for nurses to make decisions^(5)^.

Defined by a body mass index (BMI) equal to or greater than 25 kg/m², the recognition of overweight in clinical practice is commonly easy, but the proper diagnosis and identification of clinical indicators and etiologic factors still generates impasse among health professionals^(5)^.

Failure to identify overweight promptly favors the installation of the nursing diagnosis and its consequent manifestation (clinical signs and symptoms). According to research^(6)^ adolescents and young adults constitute the group most vulnerable to overweight, motivated by changes in body composition, insulin sensitivity, psychological adjustments, unhealthy eating behaviors, and the beginning of academic life. Overweight young people are often obese adults with low quality of life due to associated comorbidities and psychological diseases^(7)^.

In the care setting, the nurse and the multidisciplinary team develop measures to promote health and prevent adverse health outcomes resulting from overweight and obesity^(8)^. However, there is difficulty in raising awareness and coping with the condition on the part of clients, due, among other factors, to deficits in the professional-client approach and lack of prioritization^(9)^.

Braga et al.^(10)^ (2017) reinforce the need for nurses to have a more intriguing look at the nutritional aspects of their clientele. Nursing interventions aimed at overweight are intended to enable the population to deal with the sources of stress to restore a new, healthy and increasingly complete balance, with minimal damage caused by exposure to risk.

The literature presents studies on the prevalence of obesity and overweight in adolescents and young adults^(11, 12, 13)^. A cross-sectional study in Rio Grande do Norte, Brazil, identified a prevalence of the nursing diagnosis of overweight in 28.8% of adolescent students from public schools^(14)^. However, no studies on the development of a MRT of overweight in adolescents/young adults were identified in the literature.

The knowledge about the essential attributes, clinical antecedents, and consequences of overweight in adolescents and young adults can be used to improve the understanding of this phenomenon and nursing care. Therefore, it is considered that the clinical development of a diagnosis occurs gradually, depending on three elements: antecedent element (presumably the etiological factors that provoke the situation), consequent element (clinical indicators that represent the effects of the first element) and a set of essential attributes (key elements that define the beginning of the clinical diagnosis).

It is believed that developing a MRT of overweight will improve the diagnostic inferential process, contributing for nursing care that corresponds to the real needs of adolescents with overweight, through a theoretical-causal validation of the relationships between the diagnostic components. Furthermore, the theory can help in the improvement of the NANDA International taxonomy, as well as in the advancement of nursing science.

The research question of the study was: How is a mid-range theory, aimed at the nursing diagnosis overweight in adolescents and young adults, configured?

## OBJECTIVE

To build a mid-range theory for the nursing diagnosis overweight in adolescents and young adults.

## METHODS

To validate the concept of nursing diagnosis overweight in adolescents and young adults, a middle-range theory will be used to characterize the diagnostic structure and establish hypotheses for the clinical and causal relationships of the diagnostic components. It consists of a smaller number of more concrete concepts and analyzes their possible causal relationships, to facilitate understanding the manifestation of a phenomenon.

### Ethical aspects

Ethical review and approval were waived for this study due to not involved human beings. Declaration of Free and Informed Consent not applicable to the type of study developed.

### Design and period of study

This is a methodological study by theoretical-causal validity. This type of research aims to develop, validate and evaluate research tools and methods^(15)^. This study was developed in 2021.

The mid-range theory of overweight in adolescents and young adults was developed in light of the frameworks by Roy^(2)^ (2014) and Lopes, Silva & Herdman^(1)^ (2015). The following steps were followed: (1) definition of the approach for theory construction, (2) definition of key concepts, (3) construction of a pictorial diagram, (4) formulation of propositions, (5) establishment of causal relationships, and (6) evidence for practice.

In the first stage, an integrative literature review was carried out to present the knowledge produced about the nursing diagnosis Overweight in adolescents and young adults. This step was responsible for building the construction of the middle range theory^(1, 2)^.

Then, it was then possible to define the key concepts of the second stage: essential attributes, clinical antecedents and clinical consequences^(1)^. Conceptual and operational definitions were constructed for each key concept, with the aim of facilitating its measurement in clinical practice^(1, 2)^.

In the third stage, an illustrated pictogram-type diagram was developed with the aim of clarifying the relationships between the concepts studied. From this scheme, it was verified the relationships between concepts with greater clarity^(1)^.

The fourth stage consists of developing explanatory relationships between essential attributes, clinical antecedents and clinical consequences, based on the findings of the integrative literature review^(1)^.

The fifth stage aimed to establish relationships between two or more concepts based on evidence, provided by the results of the studies used to build the theory^(2)^. There are four categories of related factors, known as: precipitants, which are those that initiate the causal chain; predisposing factors, which act to make the individual more susceptible to a certain phenomenon; disabling, whose factors interfere with recovery and health promotion; and reinforcers, which amplify the effects of already existing conditions^(1)^.

Finally, in step six, causal relationships between key concepts were constructed, which can provide evidence for practicing and testing the constructed MRT^(1)^.

### Study protocol and sample

An integrative literature review on overweight in adolescents and young adults guided the six stages of construction of the MRT^(1, 2)^. It was developed to reveal the essential attributes, antecedents, and consequences and support the description of their conceptual and operational definitions. The synthesis of results from different studies has been considered a successful approach to creating MRTs^(2)^.

The literature review followed the framework proposed by Souza, Silva & Carvalho^(16)^ (2010) was adopted, with 6 steps: (1) elaboration of the review’s guiding questions, (2) literature search and sample selection, (3) data collection from the selected studies, (4) critical analysis of the included studies, (5) discussion of results, and (6) synthesis of the knowledge extracted from the studies.

The following guiding question was elaborated for the review: “What clinical antecedents and consequences influence the diagnosis of overweight in adolescents and young adults?”. The Person, Concept, Context (PPC) framework by Arksey & O’Malley^(17)^ (2005) was used to establish the question above.

Next, a pilot search was carried out in the literature to identify the databases with the highest number of indexed publications on the theme of overweight. The following databases were chosen: Cumulative Index to Nursing and Allied Health Literature (CINAHL), Scopus, Pubmed, and Web of Science. The database search took place between August and September 2020 and was carried out by the principal investigator. The descriptors “Overweight”, “Adolescent”, and “Young Adult” were chosen for the search, identified in MeSH and DeCS. The descriptors were paired with the Boolean operators “AND” and “OR”.

**Figure 1:**
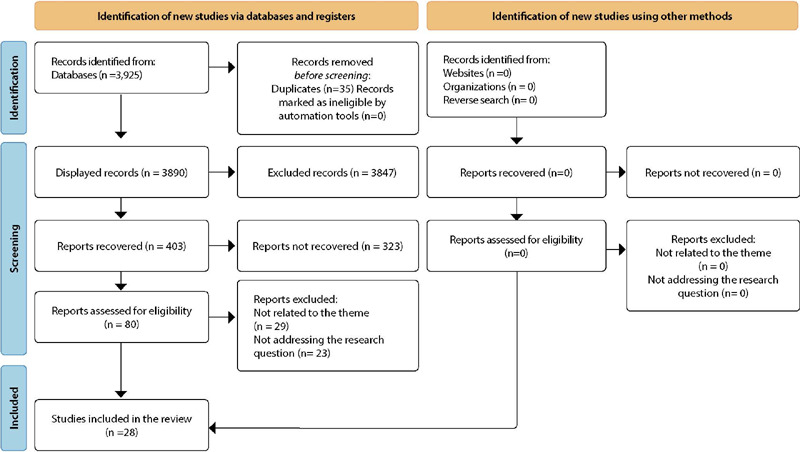
Flow chart of study selection

The inclusion criteria used to refine the searches were: free access, full-text availability, studies with target populations aged 17-24 years, and studies published from 2016 to 2020. There was no language limitation. Studies that did not respond to the research question, letters to the reader, editorials, research projects, and literature reviews were excluded. A Preferred Reporting Items for Systematic Reviews and Meta-Analyses (PRISMA) flow chart was used to present the study selection results (Figure 1).

The chosen age group (17-24 years) is in line with the World Health Organization’s^(18)^ (1986) definition of adolescence and youth – ages with a greater tendency to be overweight. The time frame (2016 to 2020) is justified by the need to list antecedents and consequences that influence the target population in recent years, since this population is impacted by temporal and daily influences.

In the third stage, the studies were categorized, and the information to be extracted from the selected articles was defined. At this stage, titles and abstracts were screened to define the sample. This step was developed through consensus between three researchers.

Next, a critical analysis of the included studies was carried out by full-text screening. The titles, authors, countries, years of publication, objectives, areas, target audiences, locations, study themes, and levels of evidence were analyzed, as well as the presence of definitions, antecedents, and consequences of overweight. This step was developed exclusively by the principal investigator.

The fifth stage included the discussion of the results from the interpretation and synthesis of the findings. Finally, the sixth stage was conducted, consisting of the final synthesis of the findings with the creation of charts and figures containing the essential attributes, antecedents, and consequences of overweight, as well as the operational and conceptual definitions.

### Analysis of the results and statistics

The data analysis was performed using the State of the Art Through Systematic Review (START) software. Studies were categorized, and the information be extracted. The results were organized into chart and figures containing the essential attributes, clinical antecedents and clinical consequences of the nursing diagnosis Overweight, as well as its operational and conceptual definitions, in order to guide the discussion. It took place also an illustrated pictogram-type diagram. The data were analyzed according to the adopted framework and related literature.

## RESULTS

Initially, 3,925 articles were found in the analyzed databases. After applying the inclusion and exclusion criteria and reading the titles and abstracts, 80 articles were selected. Despite the high number of articles initially found, the final sample consisted of 28 articles after full-text reading.

Most publications on the topic of overweight in adolescents and young adults were published in 2017 (25.0%) and 2018 (25.0%). Most studies were from Europe (32.1%), written in English (85.7%), focused on the medicine area (46.4%), and level VI evidence (71.4%).

“Intermediate body weight”, “age,” and “height” were listed as essential attributes. Thus, overweight in adolescents and young adults was defined as an “intermediate stage of increase in body weight for age and height”.

Regarding the clinical antecedents and consequences were listed based on findings from the integrative literature review, based on its incidence in adolescents and young adults^(5, 6, 19, 20, 21, 22, 23, 24, 25, 26, 27, 28, 29)^. Thirteen clinical antecedents and 7 clinical consequences were identified (Chart 1).

**Chart 1:** Clinical antecedents and consequences of overweight included in the Mid-range theories (MRT)

OVERWEIGHT	
CLINICAL ANTECEDENTS	CLINICAL CONSEQUENCES
Poor sleep quality Sedentary lifestyle Emotional disturbance Alcoholism Unhealthy eating habits Personal or family history of overweight Menarche before the age of 12 Low household income Living in urban areas Stable relationship Female sex Use of obesogenic drugs Active academic relationship	Negative self-perception of health Restrictive feeding behavior Metabolic disorders Dissatisfaction with body image Systemic arterial hypertension Mental health issues

**Figure 2:**
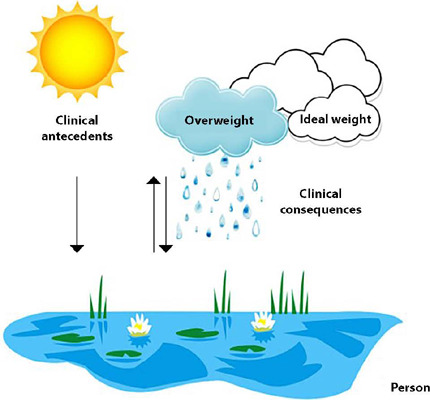
Pictogram of clinical antecedents and consequences of overweight included in the Mid-range theories (MRT)

The antecedents and consequences of overweight in adolescents and young adults were used to create a pictorial illustrated diagram. This pictogram was based on the rain cycle to trace the influence of clinical antecedents that predispose to the installation of overweight and its clinical manifestation through the consequences (Figure 2).

Water represents the human being adolescent or young adult individual. This individual, like water, is subject to internal and external stimuli that cause a change in its initial physical state. A frequently observed case is the rain cycle. In this cycle, water needs the influence of the sun to evaporate, in the same way that the individual develops a relationship with the clinical history of excess weight. The cloud, in the pictogram, is responsible for holding a factor that was not common to it (water), in a similar way to how an individual holds excess weight. When this occurs, the cloud manifests its change through rain (clinical consequences), this being the final stage of the cycle. It is noteworthy that the final phase of the rainy cycle and the clinical consequences of excess weight are easier to detect during the establishment of health interventions.

In this sense, to explain overweight in adolescents and young adults, it was considered that the individual (water) is influenced by several stimuli (the sun) that can cause the occurrence of a condition overweight (the cloud), manifested through clinical consequences (the rain). Therefore, clinical antecedents and clinical consequences act cyclically on the individual, impacting their relationship with overweight.

Next, 11 propositions were elaborated to elucidate the causal relationships between the clinical antecedents and consequences of overweight, as shown below.

1. The finding of overweight in the clientele will depend directly on the individual’s body weight, age, and height.
2. Etiological factors of overweight can be divided into modifiable and non-modifiable. The first axis encompasses poor sleep quality, sedentary lifestyle, and unhealthy eating habits. The second includes factors such as personal or family history of overweight, menarche before the age of 12, and female sex.
3. External factors such as active academic relationship and emotional disturbance directly interfere with weight gain in adolescents and young adults, as the intensity of daily activities leads this clientele to consume high-calorie and easily accessible foods.
4. The modernization process directly influences overweight rates in the population since factors such as living in an urban area and having a low household income are clinical antecedents of overweight in adolescents and young adults.
5. The modernization process directly influences overweight rates in the population since factors such as living in an urban area and having a moderate household income are clinical antecedents of overweight in adolescents and young adults.
6. The individual’s lifestyle directly impacts the predisposition to overweight. The presence of factors such as sedentary lifestyle, alcoholism, inadequate lifestyle habits and use of obesogenic drugs (clinical antecedents) justifies this fact.
7. The family environment and personal history directly influence the predisposition to be overweight since offering foods with high caloric content and low nutritional value at home generates unhealthy eating habits and excess weight.
8. One of the main consequences of overweight is mental health issues, especially in the female universe.
9. Adolescents and young adults with overweight tend to be dissatisfied with their body image, as they overvalue the view of a thin body and seek social acceptance. This fact is justified by the neuropsychological development of this stage of life and leads them to adhere to restrictive weight control behaviors, sometimes putting their health at risk.
10. Metabolic disorders, especially dyslipidemia, diabetes mellitus, and increased blood pressure are some clinical manifestations of excess weight in adolescents and young adults that deserve special attention, as they increase the risk of premature death.
11. Overweight adolescents and adults negatively perceive health, as they associate an overweight body with a non-aesthetically pleasing and clinically ill body.

Finally, 12 causal relationships involving the etiological factors were established according to Lopes, Silva & Herdman’s^(1)^ (2015) framework. Thus, the present MRT has 3 precipitating factors: sedentary lifestyle, unhealthy eating habits, and poor sleep quality, 3 predisposing factors: emotional disturbance, personal or family history of overweight, and active academic relationship, 2 disabling factors: alcoholism and use of obesogenic drugs, and 5 reinforcing factors: menarche before the age of 12, low household income, living in urban areas, stable relationship, and female sex.

## DISCUSSION

Nursing is constantly developing knowledge to understand the changing health needs of individuals and the community^(2)^. The development of MRTs is observed in different branches of nursing: health education^(30)^, chronic pain^(31)^, mental health^(32)^, intensive care^(33)^, death and dying^(34)^. However, the absence of studies on the nursing diagnosis overweight, and in the population of adolescents and young adults is highlighted.

The MRT developed in the present study was performed with a final sample of 28 articles. It is important to highlight that no studies were found that addressed the theme of the nursing diagnosis overweight and the development of a MRT, demonstrating that these studies are still incipient. This reality is a current problem and corroborates with previous research in different areas^(35, 36, 37)^, as the lack of studies makes it difficult for new researchers to make the method robust and reliable.

Among the studies focused on the theme of overweight, it was noticed that most publications were from the medicine area to the detriment of nursing, with most studies coming from the European continent and being written in English. This fact is believed to be related to the high costs of research in the health area and the greater funding for research that this field and continent receive, which explains the review’s findings^(35)^.

Regarding the study designs, cross-sectional studies stood out (level VI evidence). Melnyk & Fineout-Overholt^(38)^ (2011) state that the level of evidence from these studies is not strong, denoting the need for more rigorous studies.

The etiology of overweight is quite complex and has a multifactorial nature, involving biological, psychological and socioeconomic factors. It is known that the profound changes that have occurred in society, such as urbanization and the increase in production and consumption of processed foods, causing a sharp increase in sedentary lifestyle and consumption of ultra-processed foods, have contributed to the increase in overweight and obesity on a global scale^(39)^. These findings corroborated the clinical antecedents and clinical consequences listed in the present research.

Therefore, the consumption of foods with high energy density and rich in lipids and simple carbohydrates alone cannot explain the exponential increase in this clinical condition, and it is essential to investigate other behavioral and socioeconomic factors, such as the rate of physical activity and income. individual’s family^(40)^.

A relevant association that can be made is that overweight in adolescents and young adults is directly related to the lack of physical activity. Recent studies state that the high amount of time dedicated to low-intensity activities, such as watching television, using the computer and playing video games, is directly related to the increased risk of being overweight among adolescents and young adults^(41, 42)^.

It should also be noted that encouraging excessive wakefulness, based on social and recreational life, early school hours, academic tasks and family demands, can lead to fatigue and a direct reduction in physical activity levels. The lower energy expenditure resulting from poor sleep quality facilitates the accumulation of body fat^(43)^.

Regarding socioeconomic factors, an increase in overweight is observed in low-income individuals living in urban regions. Within the criteria for food consumption in the group, there was a close relationship between food and socioeconomic conditions, which is often guided by the monetary value of foodstuffs. In other words, the price of the items defines the food selection^(22)^.

In this context, food underconsumption occurs, with the search for a feeling of satiety and the fight against hunger through the consumption of bread and sugary infusions. Poor living conditions require the selection of items with high calorie content and low nutritional value^(44)^.

In addition to socioeconomic and behavioral factors, there are also biological factors. In this aspect, female sex or menarche before the age of 12 stands out. The accumulation of fat in the female population is mainly due to the secretion of sex hormones that increase adiposity and appetite. The earlier the exposure to estrogen and other adrenal steroids, the greater the chance of being overweight^(19)^.

As for psychological factors, the role of food as an element of comfort for overcoming dilemmas, tensions and daily responsibilities stands out. Inadequate appetite control and binge eating induced by daily stress seem to make it difficult to control body weight^(5)^.

Once in possession of these symptoms, the individual tends to express guilt for not reaching the desired weight and, on a large scale, psycho-emotional disorders. Among the main consequences of excess weight on the psychological health of adolescents and young adults, the impairment of mental health stands out, evidenced by negative self-perception of health, dissatisfaction with body image and abuse of restrictive diets^(19, 20)^.

Thus, given the multicausal nature of excess weight, the role of the professional nurse as a conciliator between the health demands and the individual’s daily life stands out, based on specialized and targeted consultations. History taking and physical examination are essential to prevent or identify excess weight, as well as plan care, evaluating the client’s signs and symptoms Health promotion stands out, with the development of health education at school, as a form of intervention^(45)^.

Furthermore, the development of MRT of the nursing diagnosis will provide nurses with greater knowledge about the main concepts of this phenomenon, which are the attributes, antecedents and consequences. It is also important to highlight the existence of theoretical propositions and the possibility of them being tested empirically. All of this contributes to bringing theory and practice closer together.

### Study limitations

As a limitation of this study, we can point out the use of only open access articles in the integrative literature review, which may have contributed to the small sample size.

### Contributions to nursing knowledge

This middle-range theory makes an important contribution to furthering the research on overweight in adolescents and young adults, since it provides a useful tool for nursing professionals to efficiently detect signs and symptoms on clinical practices. Therefore, the results of this theory can provide the basis for moving forward with new stages of research to empirically prove the identified concepts, in order to proceed with validation of content and clinical validation.

## CONCLUSION

The construction of the mid-range theory of overweight in adolescents and young adults was based on 28 articles from an integrative literature review. Three essential attributes, 13 antecedents, and 7 consequences of the studied nursing diagnosis were identified, which were conceptually and operationally defined. An illustrated pictogram was created to facilitate understanding, demonstrating the influence of these indicators. Furthermore, 12 propositions and 13 causal relationships were created.

The development of a MRT based on the nursing diagnosis overweight proved useful and able to guide interventions to change unhealthy conditions and favor nursing care, reinforcing the cyclical nature of the disease. This theoretical-causal validity can be used by nurses to confirm nursing diagnosis overweight in adolescents and young adults.
